# Comprehensive analysis of molecular immunological characteristics and potential biomarkers in brucellosis

**DOI:** 10.3389/fimmu.2026.1614025

**Published:** 2026-02-04

**Authors:** Rui Wang, Juan He, Xiao Li, Yue Shi, Huijuan Duan, Haitao Ding

**Affiliations:** 1 Clinical Laboratory Medicine Center, Inner Mongolia People’s Hospital, Hohhot, Inner Mongolia, China; 2 Inner Mongolia Academy of Medical Sciences, Hohhot, Inner Mongolia, China

**Keywords:** bioinformatics, biomarkers, brucellosis, differential expression analysis, machine learning, weighted gene co-expression network analysis (WGCNA)

## Abstract

**Objective:**

This study aims to explore potential biological biomarkers for brucellosis by integrating transcriptomic profiling and bioinformatics-driven approaches.

**Methods:**

Differentially expressed genes (DEGs) associated with acute and chronic brucellosis were identified using transcriptomic data from the Gene Expression Omnibus (GEO). Functional annotation and pathway enrichment analysis of DEGs were performed using Gene Ontology (GO) and the Kyoto Encyclopedia of Genes and Genomes (KEGG). Weighted Gene Co-expression Network Analysis (WGCNA) was applied to construct gene co-expression modules, followed by screening of modules significantly correlated with disease phenotypes. Notably, a multi-model machine learning framework was employed for systematic screening, cross-validation, and validation of diagnostically relevant biomarkers—ensuring robustness and generalizability of the findings.

**Results:**

A total of 103 brucellosis patients and 46 healthy controls with whole blood transcriptomic profiles were included. Comparative analysis identified 264 DEGs, which were predominantly enriched in mitotic nuclear division, chromosome segregation, nucleocytoplasmic transport, cell cycle regulation, and cytokine-cytokine receptor interaction pathways—providing novel insights into the molecular pathogenesis of brucellosis. Immune infiltration profiling revealed that brucellosis progression was positively correlated with CD8+ T cells, follicular helper T cells, and resting NK cells—highlighting previously underappreciated immune regulatory mechanisms. Two co-expression modules were significantly associated with brucellosis clinical traits through WGCNA. Cross-validation using machine learning algorithms (LASSO, SVM, random forest) prioritized six overlapping hub genes: RTP5, KIF19, CDKN2A, RCAN2, GLB1L3, and IL12RB2. Receiver Operating Characteristic (ROC) curve analysis demonstrated robust diagnostic performance, supporting their potential as combinatorial biomarkers for brucellosis detection.

**Discussion:**

These novel hub genes are closely implicated in inflammatory responses, neutrophil regulation, and B cell receptor signaling pathways—key processes underlying brucellosis pathogenesis that have not been previously targeted for diagnostic biomarker development. This work not only enhances our understanding of brucellosis biology but also lays a critical foundation for the development of non-invasive, accurate diagnostic tools and targeted therapeutic strategies—filling a significant gap in current brucellosis management.

## 1 Introduction

Brucellosis is a highly contagious intracellular bacterial disease caused by the genus *Brucella*. Its typical symptoms include fever (87%), fatigue (63%), arthralgia (62%), and myalgia (56%) (1). Recent epidemiological surveillance indicates a substantial upward revision in global brucellosis incidence rates, with annual case estimates now ranging from 1.6 to 2.1 million new infections (2, 3). The international prevalence of this disease is primarily concentrated in the Mediterranean coastal regions, South America, Africa, and Asia, particularly in Western Asia, including Syria, Iraq, Turkey, and Iran, as well as North Africa, specifically Egypt (4). In China, human brucellosis ranks fifth among all notifiable infectious diseases in terms of case numbers. In 2021, 69,767 cases of brucellosis were reported, with an incidence rate of 4.95/100,000 (5). Brucellosis, therefore, remains a significant public health concern (6) and poses a substantial threat to human health and socioeconomic development, placing continued strain on the public health system.

Brucellosis manifests in both acute and chronic forms, with clinical presentations that are diverse and often overlap with other febrile illnesses (e.g., typhoid fever, rheumatic fever, and septic arthritis). This syndromic mimicry poses a significant diagnostic challenge, as the nonspecific constellation of symptoms frequently leads to misdiagnosis and subsequent delays in appropriate therapy (7, 8). The diagnostic approach for brucellosis encompasses direct methods, such as bacteriological isolation and molecular confirmation, and indirect methods, including *in vitro* serological assays and *in vivo* hypersensitivity testing. Although bacteriological isolation of *Brucella* spp. from sterile body fluids or tissues remains the diagnostic gold standard (typically performed using automated blood culture systems), its clinical utility is limited by the requirement for prolonged incubation periods and the relatively low positive results. During the initial febrile phase, frontline serological screening relies on the Rose Bengal Test (RBT), Serum Agglutination Test (SAT), and Complement Fixation Test (CFT) (9). However, these conventional assays carry inherent cross-reactivity risks with commensal *Brucella*-like organisms, frequently generating false-positive results and lacking sufficient diagnostic accuracy across different disease phases (10). Given these limitations, a major challenge for future research is the identification of novel diagnostic and prognostic biomarkers (11). Key focus areas include: (I) developing and validating novel diagnostic approaches to identify potential biomarkers, thereby reducing reliance on blood cultures; (II) creating more effective vaccines to protect animals and professional populations; and (III) establishing whole-genome sequencing (WGS) to enable real-time outbreak delineation, zoonotic source attribution, transmission bottleneck identification, and detection of emergent hypervirulent clones.

Transcriptomics serves as a pivotal tool in the research and diagnosis of brucellosis. By analyzing the transcriptome of specific cells or tissues during Brucella infection, encompassing mRNA and non-coding RNA, it is possible to deeply uncover the pathogenic mechanisms and regulatory processes involved in the infection (12). In mRNA-related research, Mitroulis et al. (13) were the first to conduct in depth analysis of transcriptomic alterations in human phagocytes during *Brucella* infection, as well as in peripheral blood immune populations during active disease, thereby constructing a gene map of the immune response in human Brucella-host interactions. Wang et al. (14) employed single-cell RNA sequencing (scRNA-seq) to comprehensively analyze the immune cell landscape of peripheral blood from 29 brucellosis patients cross different disease stages. Their findings revealed significant changes in the proportion and function of immune cells, presenting diverse immune characteristics of brucellosis and deciphering the core role of immune cells in resisting *Brucella* infection. The critical roles of mRNAs in microbial infections have been progressively elucidated, with accumulating evidence indicating their pivotal involvement in pathogen transmission and pathogenic mechanisms (15, 16). Pathogens can modulate host mRNA expression, and differentially expressed mRNAs may serve as auxiliary diagnostic indicators for bacterial diseases (17, 18). Therefore, we hypothesize that alterations in whole blood mRNA profiles may provide potential biomarkers for *Brucella* infection, representing a promising novel direction in brucellosis research. In this study, we retrieved the dataset “GSE69597” from the Gene Expression Omnibus (GEO) database using the search term “brucellosis”, which initially consisted of 169 samples. Following the exclusion of 17 patients with Leishmania infection, 2 cases with incomplete data pertaining to brucellosis, and 1 healthy control subject, a total of 149 participants were included in the final analysis. This cohort comprised 75 patients with acute brucellosis, 28 patients with chronic brucellosis, and 46 healthy controls. Through analysis of gene expression profiles, we identified 264 differentially expressed genes (DEGs). Weighted Gene Co-expression Network Analysis (WGCNA) revealed two modules significantly associated with clinical traits. Based on these analytical results, six hub genes were identified. To further explore the potential diagnostic value of these genes for brucellosis, we performed machine learning-based classification modeling and biological enrichment analysis.

## 2 Methods

### 2.1 Data filtering and standardization

The dataset with the accession number GSE69597 was downloaded from the GEO database. The raw data of this dataset are Gene Counts, which were converted to TPM (Transcripts Per Kilobase of exon model per Million mapped reads) values using R language. Subsequently, pseudogenes and lncRNAs (long non-coding RNAs) were removed to retain transcriptomic mRNA data. Genes with a proportion of zero expression values greater than 50% across samples were excluded. Finally, we obtained an mRNA gene expression matrix containing 14,382 genes and 149 samples, which was used for subsequent bioinformatics analysis.

### 2.2 Gene expression analysis of whole blood

To acquire the mRNA expression matrix, we retrieved the dataset GSE69597 from the GEO database using the keyword “brucellosis.” Differential expression analysis was conducted in R using the “limma” package (version 3.56.2), comparing 103 brucellosis patients and 46 healthy controls, with thresholds of *P* < 0.05 and |log_2_FC| ≥1. To visualize the upregulated and downregulated DEGs, we utilized the R package(version 3.4.4) “ggplot2” and “pheatmap” package (version 1.0.12) in R. Additionally, functional enrichment analysis of DEGs was performed via Gene Ontology (GO) and Kyoto Encyclopedia of Genes and Genomes (KEGG) annotations to elucidate their biological functions and associated signaling pathways.

### 2.3 Correlation analysis of immune infiltration

The CIBERSORT algorithm was used to estimate the proportions of 22 immune cell subsets in 149 whole blood samples. Visualization of immune cell proportion distributions was achieved via stacked bar plots generated using the R package “ggplot2”. Subsequently, the differentially abundant immune cell subsets were then used as trait variables for subsequent WGCNA.

### 2.4 WGCNA

WGCNA was performed using the R package “WGCNA” (version 1.72.1) to construct scale-free co-expression networks and explore associations with clinical traits. Initially, hierarchical clustering was conducted to identify and filter outlier samples, resulting in the exclusion of sample SP_CT2. Subsequently, an optimal soft-thresholding power (β) was determined to construct a weighted adjacency matrix, yielding a scale-free topology fit index of R^2 = 0.9. Dynamic tree cutting was then applied to identified 12 distinct co-expression modules, each assigned a unique color code for categorization and labeling. The adjacency matrix was subsequently transformed into a topological overlap matrix (TOM), with module assignments annotated by color labels and module eigengenes (ME). Finally, Pearson correlation analysis was applied to evaluate the relationships between MEs and clinical traits.

### 2.5 Shared genes and correlation analysis

Genes with high intramodular connectivity, defined as hub genes per WGCNA nomenclature, were identified as key candidates. Cytoscape software (version 3.9.1) was used to visualize the interaction networks of these hub genes. Functional enrichment analysis, including GO and KEGG annotations, was conducted on the genes within the models to elucidate their associated biological processes and signaling pathways. Venn diagram was utilized to depict the overlapping genes between WGCNA-derived hub genes and DEGs. Spearman’s rank correlation analysis was performed to assess correlations among the overlapping genes, while box plots were utilized to explicitly display their expression levels across the acute brucellosis, chronic brucellosis, and healthy controls.

### 2.6 Biomarker screening, diagnostic efficacy and functional correlation analysis

Candidate biomarkers were identified via multiple machine learning algorithms including LASSO regression, Gaussian mixture model (GMM), and Recursive Feature Elimination-Support Vector Machine with Radial Basis Function (SVM-RBF). To determine the final biomarkers, Venn diagrams were employed to visualize the overlapping genes among the outputs of the three algorithms. The diagnostic efficacy of the selected genes was assessed by constructing receiver operating characteristic (ROC) curves based on their expression profiles. Additionally, correlations between candidate genes and immune cells were analyzed. A nomogram model was established to facilitate diagnosis of brucellosis based on these candidate biomarkers, and its diagnostic performance was further validated via decision curve analysis, implemented using the R package “rms”. miRNA-mRNA regulatory networks were constructed by predicting miRNAs targeting the candidate genes, with data integrated from three authoritative databases (TargetScan, miRTarBase, and miRDB), and visualized using Cytoscape software. Furthermore, transcription factors (TFs) regulating the key candidate mRNAs were predicted using ENCODE data.

## 3 Results

### 3.1 Differential gene screening and functional analysis between brucellosis and healthy controls

**Figure 1:**
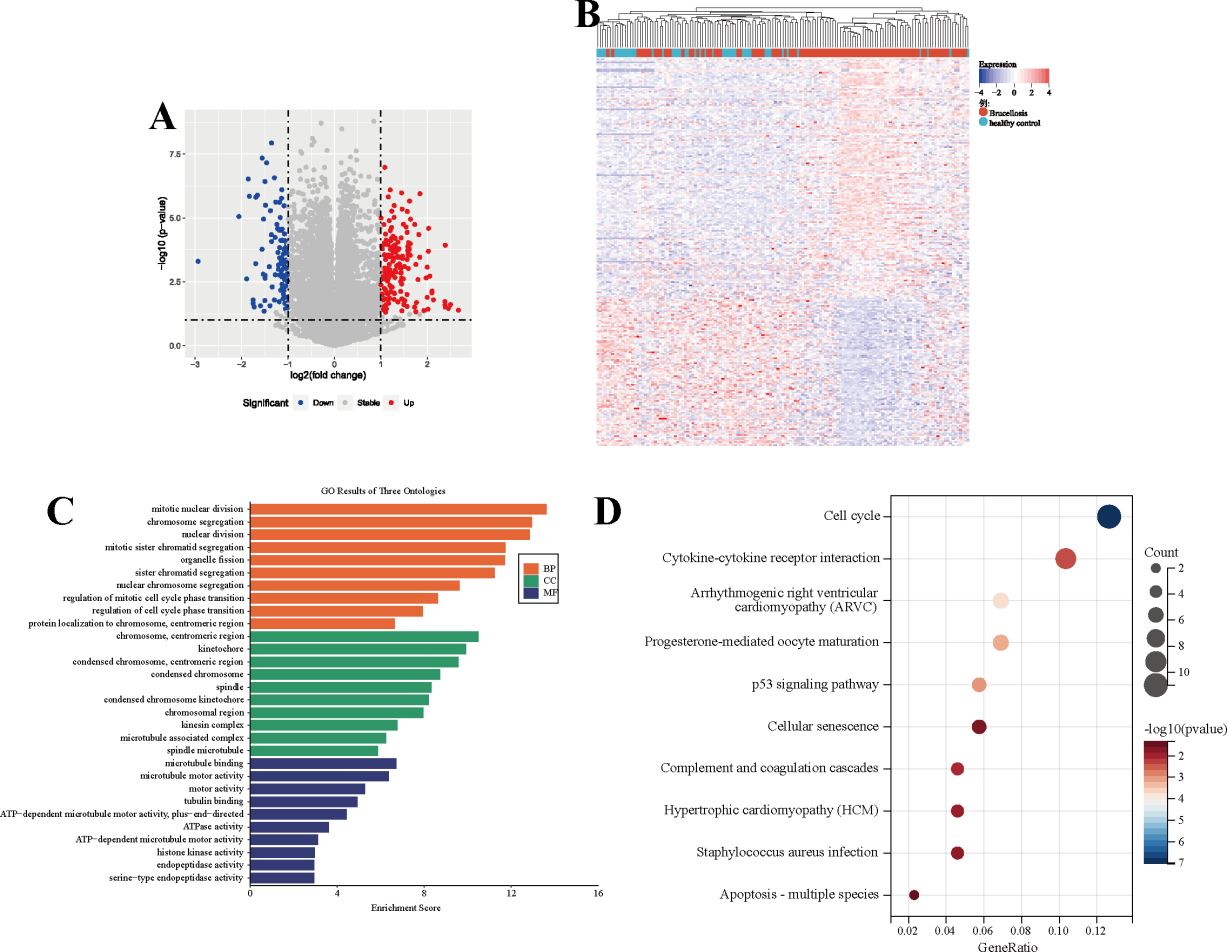
Differential gene screening and functional analysis between brucellosis and healthy controls. **(A)** Volcano plot shows the differential protein expression between the brucellosis group and the healthy control group. **(B)** Heat map of differentially expressed genes between brucellosis group and healthy control group. **(C)** Gene Ontology (GO) analysis of differentially expressed genes. **(D)** Kyoto Encyclopedia of Genes and Genomes (KEGG) analysis of differentially expressed genes.

Collectively, this study analyzed mRNA profiles from 103 brucellosis patients and 46 healthy controls, leading to the identification of 264 DEGs, including 163 upregulated and 101 downregulated genes. A volcano plot was used to visualize the distribution of these DEGs (**Figure 1A**), while detailed expression patterns of the upregulated and downregulated genes were illustrated in a heatmap (**Figure 1B**). GO enrichment analysis demonstrated significant enrichment of DEGs in biological processes, such as mitotic nuclear division, chromosome segregation, and nucleocytoplasmic transport. Additionally, molecular functions including microtubule binding, microtubule motor activity, and tubulin binding, as well as cellular components such as chromosomal centromeric regions, kinetochores, and condensed chromosome centromeres, were significantly represented (**Figure 1C**). Furthermore, KEGG pathway analysis indicated that DEGs were significantly associated with key signaling pathways, including the cell cycle regulation, cytokine-cytokine receptor interaction, p53 signaling pathway, and Staphylococcus aureus infection (**Figure 1D**). Collectively, these results suggest that the DEGs identified could serve as potential diagnostic biomarkers for brucellosis.

### 3.2 Correlation between brucellosis and immune infiltration

**Figure 2:**
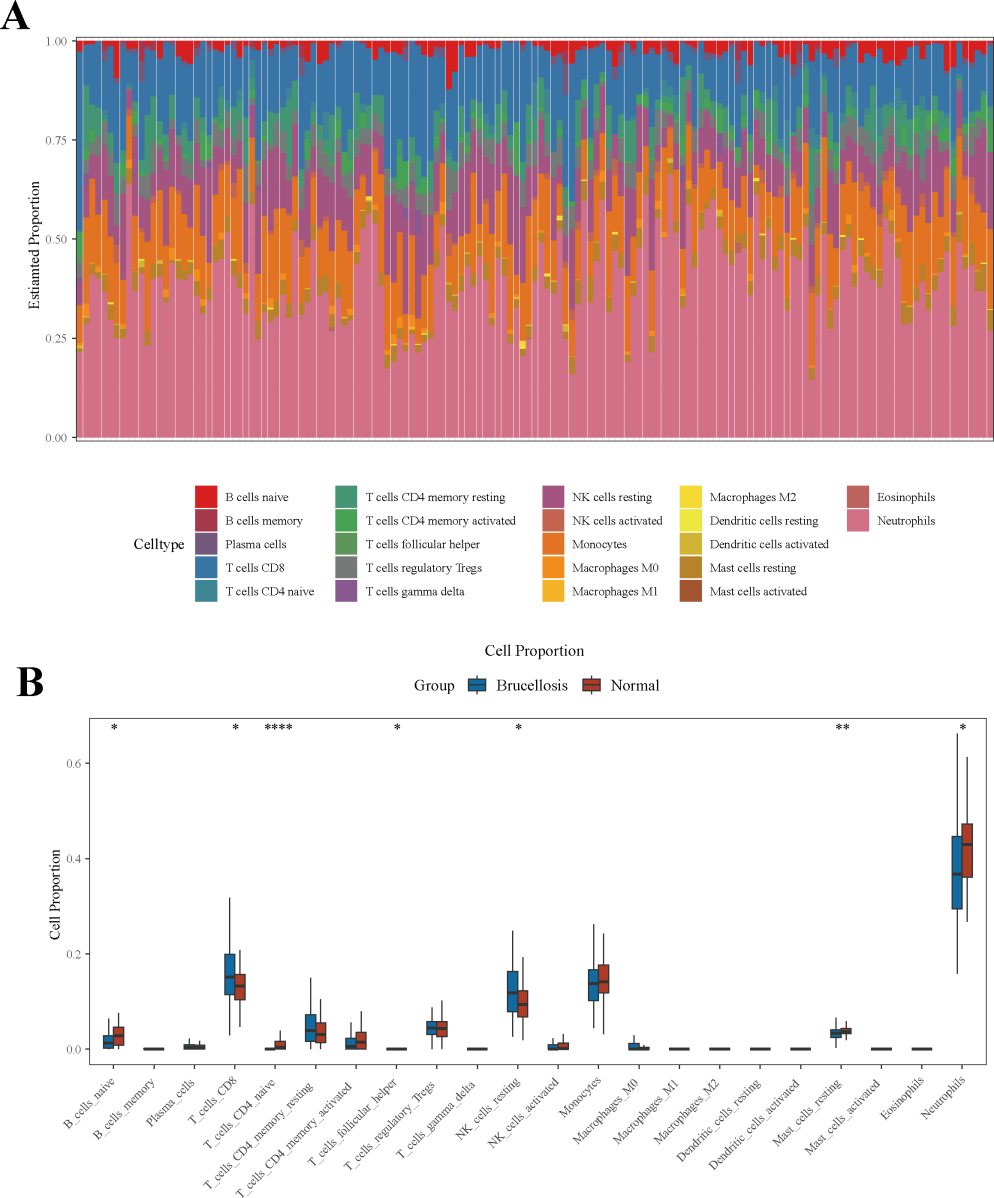
Correlation analysis of brucellosis and immune infiltration. **(A)** accumulation plot of the proportion of 22 immune cells in 149 whole blood samples. **(B)** Box plots of immune cells in the brucellosis group and healthy controls.

To dissect the immunological landscape underlying brucellosis pathogenesis, we analyzed correlations between whole-blood transcriptomic profiles and immune cell subsets distributions, with the latter visualized via a stacked bar plot (**Figure 2A**). Our results revealed significant differences in immune cell proportions between the brucellosis group and healthy controls. Specifically, the brucellosis group exhibited significantly higher proportions of CD8^+^ T cells, T follicular helper cells, and resting NK cells (*P* < 0.05). In contrast, there were reduced frequencies of naïve B cells, naïve CD4^+^ T cells, resting mast cells, and neutrophils in the brucellosis group (*P* < 0.05) (**Figure 2B**). These findings suggest a significant correlation between the progression of brucellosis and the dysregulation of specific immune cell subsets.

### 3.3 Co-expressed gene modules in brucellosis patients and their correlation with clinical features

**Figure 3:**
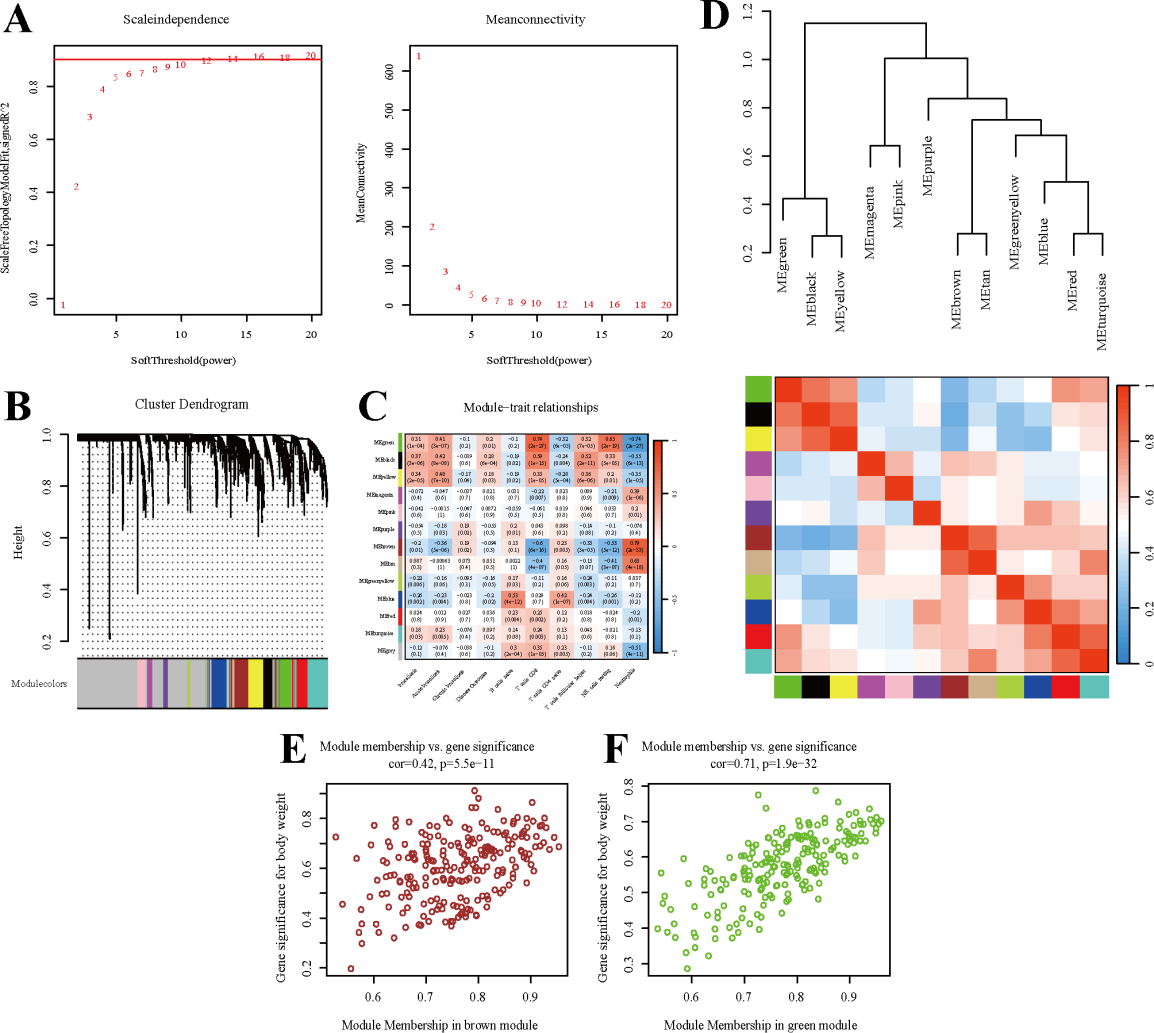
Identification of co-expressed protein modules using WGCNA. **(A)**β=12 was selected to establish a scale-free network. **(B)** Dendrogram of clustering. **(C)** Heatmap showing the correlation between gene modules and clinical features. **(D)**Inter-module correlation analysis. **(E, F)** Correlation of the two most highly correlated modules.

To identify co-expressed gene modules, we constructed a weighted co-expression network using WGCNA, resulting in 12 eigengene modules (MEs) as shown in **Figures 3A, B**, with the optimal soft-thresholding power being selected as β = 12. To explore the biological relevance of these modules, we analyzed correlations between module eigengenes and clinical traits, as illustrated in **Figure 3C**. The brown module (221 genes) exhibited a significant positive correlation with neutrophils (r = 0.79, *P* < 0.001), while the green module (206 genes) showed a positive correlation with CD8^+^ T cells (r = 0.74, *P* < 0.001) and a negative correlation with neutrophils (r = -0.74, *P* < 0.001), a pattern that aligns with the well-established antagonistic crosstalk between adaptive and innate immunity. Furthermore, inter-module correlation analysis (**Figure 3D**) revealed positive associations between the green module and black/yellow/red/cyan modules, but negative correlations with other modules, most prominently the brown module. Finally, scatter plots (**Figures 3E, F**) demonstrated strong positive correlations between intramodular connectivity and gene significance (GS) values for both brown and green modules, with high concordance confirming the biological relevance of hub genes to clinical phenotypes.

### 3.4 Identification and classification of key genes associated with Brucellosis and enrichment analysis of biological functions

**Figure 4:**
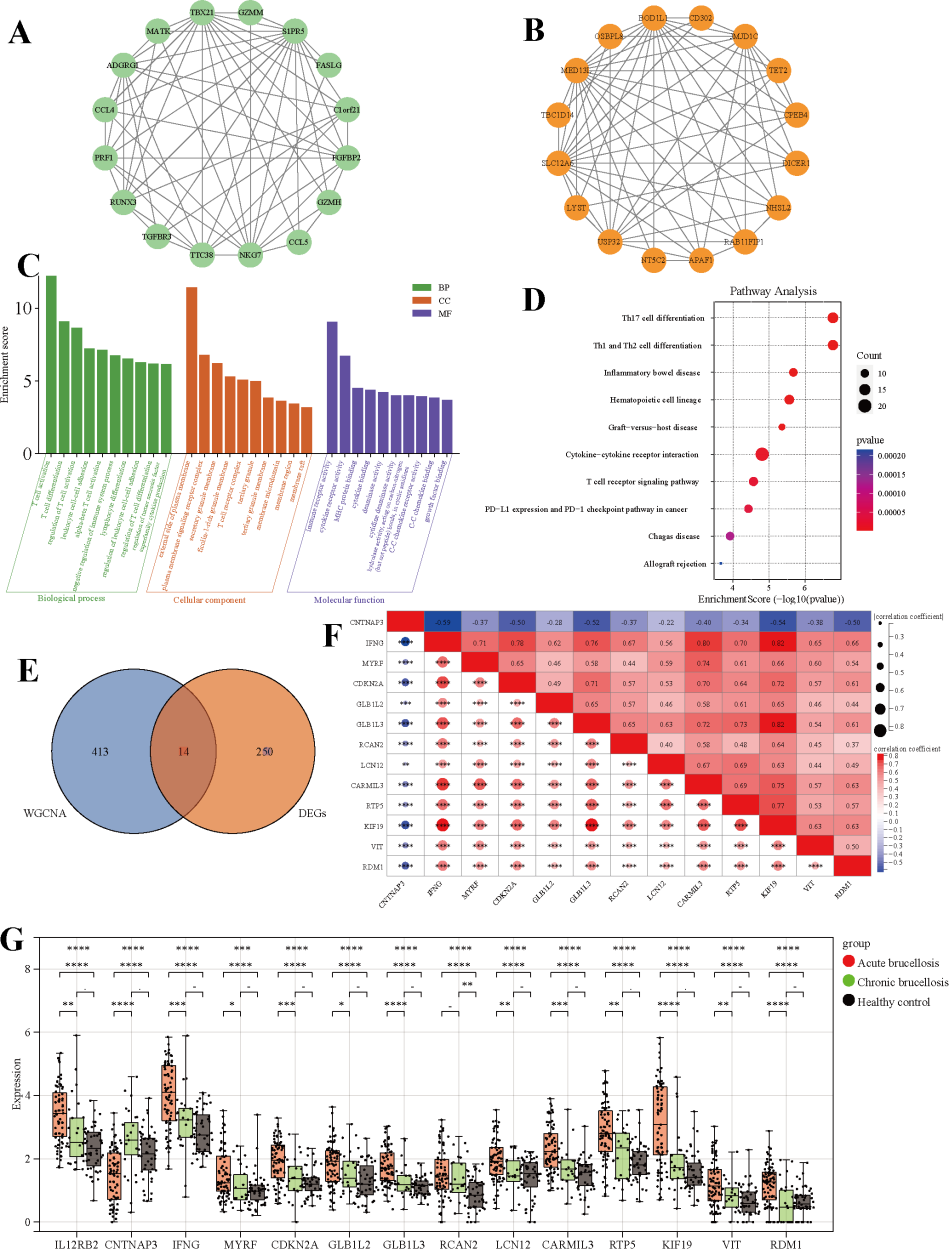
Identification and classification of key genes associated with Brucellosis and enrichment analysis of biological functions. **(A, B)** Interaction network diagram of the top 16 genes in the green and brown modules. **(C)** Gene Ontology (GO) analysis of the green and brown module genes. **(D)** The Kyoto Encyclopedia of Genes and Genomes (KEGG) analysis of the green and brown module genes. **(E)** Wenn diagram of the key genes in the intersection of WGCNA and DEGs. **(F)** Heatmap of the correlation analysis of the 14 key genes. **(G)** Box plots of key genes in the acute and chronic groups of brucellosis and healthy controls.

Genes with higher module membership (ME) exhibited stronger correlations with clinical traits. To explore gene interaction networks, the top 16 hub genes from the green (**Figure 4A**) and brown (**Figure 4B**) modules were visualized using Cytoscape (v3.9.1). Functional enrichment analysis of 427 genes within these two modules was performed based on Gene Ontology annotations (**Figure 4C**), highlighting several key biological processes and molecular features. In the category of Biological Processes, the genes were enriched in processes involved in the regulation of T cell activation and differentiation, leukocyte-cell adhesion, lymphocyte differentiation, and negative regulation of immune responses. For Molecular Functions, significant terms included immune receptor activity, cytokine receptor activity, MHC protein binding, and cytokine binding. Cellular Component analysis identified associations with the external side of the plasma membrane, plasma membrane signaling receptor complexes, secretory granule membranes, and T cell receptor complexes. Complementary KEGG pathway enrichment analysis (**Figure 4D**) revealed significant associations with Th17 cell differentiation, Th1/Th2 cell differentiation, hematopoietic cell lineage, inflammatory bowel disease, cytokine-cytokine receptor interactions, and T cell receptor signaling pathways. Furthermore, Venn diagram-based intersection analysis between WGCNA-derived hub genes and DEGs identified 14 overlapping hub genes (**Figure 4E**). Spearman’s rank correlation analysis demonstrated that CNTNAP3 showed negative correlations with the other 13 genes, whereas the remaining 13 genes displayed positive intercorrelations (**Figure 4F**). Finally, box plot visualization of the expression levels of these overlapping genes across acute-phase, chronic-phase, and healthy control groups (**Figure 4G**) revealed that all genes except CNTNAP3 displayed elevated expression in brucellosis patients. Notably, gene expression levels were higher in acute-phase patients compared to their chronic-phase counterparts.

### 3.5 Screening and comprehensive correlation analysis of potential biomarkers for brucellosis

**Figure 5:**
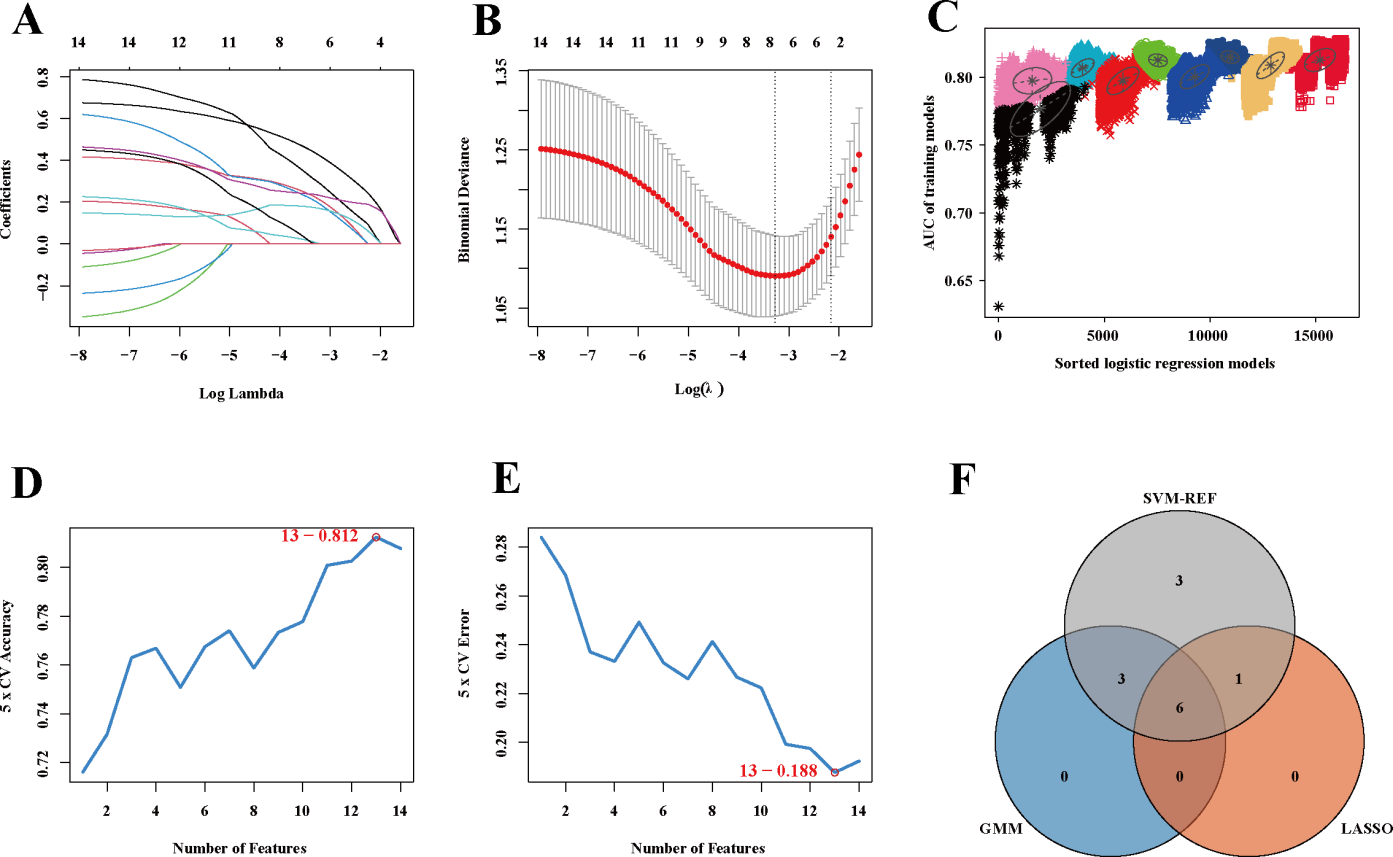
Machine Learning Algorithm Selection of Potential Biomarkers for Brucellosis Diagnosis. **(A, B)** The LASSO regression extracted the key gene map. **(C)** Plot of key genes extracted by GMM. **(D, E)** SVM-REF to extract the key gene map. **(F)** Wenn diagram of screening key genes by machine learning methods.

**Figure 6:**
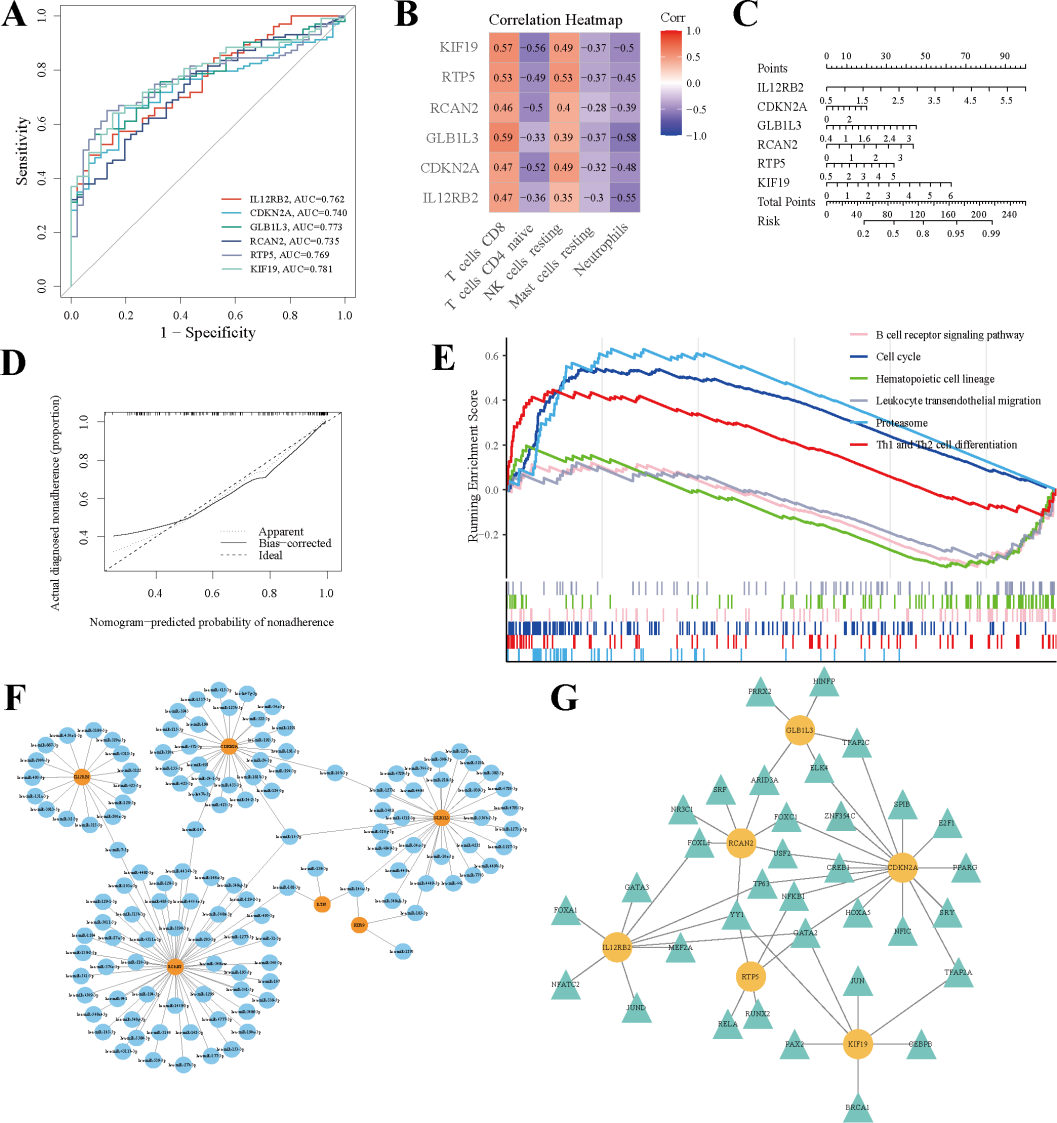
Diagnostic efficacy and immune correlation analysis of potential biomarkers in brucellosis. **(A)** Diagnosis of ROC for key genes. **(B)** Heatmap of the correlation between key genes and immune cells. **(C)** The nomogram model diagram. **(D)** The decision plot. **(E)** Single-gene enrichment analysis. **(F)** Predict network graph of the target genes. **(G)** Transcription factor interactions plot for the predicted key genes.

To identify and prioritize potential diagnostic biomarkers from the 14 overlapping genes, three complementary machine algorithms approaches were employed to ensure rigorous and reliable selection. LASSO regression analysis identified 7 candidate biomarker genes (**Figures 5A, B**), while Gaussian Mixture Model (GMM) clustering uncovered 9 biomarker-associated genes (**Figure 5C**). Additionally, Support Vector Machine-Recursive Feature Elimination (SVM-RFE) highlighted 13 potential biomarkers (**Figures 5D, E**). Subsequently, intersection analysis of the outputs from these algorithms revealed 6 consensus genes—RTP5, KIF19, CDKN2A, RCAN2, GLB1L3, and IL12RB2—representing highly reliable biomarker candidates for brucellosis diagnosis (**Figure 5F**). ROC curves (**Figure 6A**) demonstrated robust diagnostic efficacy for each gene: RTP5 (AUC = 0.762), KIF19 (AUC = 0.740), CDKN2A (AUC = 0.773), RCAN2 (AUC = 0.735), GLB1L3 (AUC = 0.769), and IL12RB2 (AUC = 0.781), validating their potential as valuable biomarkers. Correlation analysis (**Figure 6B**) revealed positive associations between these six genes and CD8^+^ T cells/resting NK cells, while negative correlations were observed with naïve CD4^+^ T cells, mast cells, and neutrophils. The diagnostic nomogram (**Figure 6C**) and decision curve analysis (**Figure 6D**) further confirmed the superior clinical utility of the combinatorial biomarker panel. Single-gene set enrichment analysis (GSEA) indicated significant enrichment (adjusted *P* < 0.05) in pathways including the B cell receptor signaling, cell cycle, hematopoietic cell lineage, leukocyte transendothelial migration, proteasome, and Th1/Th2 differentiation (**Figure 6E**). Additionally, a preliminary regulatory network was constructed, integrating the six mRNAs with 131 predicted targeting miRNAs (**Figure 6F**). Transcription factor (TF) prediction based on ENCODE ChIP-seq data identified 34 TFs potentially regulating these mRNAs (**Figure 6G**).

## 4 Discussion

In this study, we integrated transcriptomic profiling and advanced bioinformatics analyses to perform comprehensive, in-depth investigations of whole blood samples from brucellosis patients (encompassing both acute and chronic phenotypes) and healthy controls. Significant gene expression alterations were detected, which were strongly associated with immune-related pathways. Six hub DEGs were identified as potential diagnostic or prognostic biomarkers for brucellosis. Furthermore, the molecular characteristics of these genes may provide valuable insights into the underlying mechanisms and pathological processes of brucellosis.

Consistent with the critical role of key gene identification in advancing brucellosis research (19), this study employed two well-established bioinformatics methods: differential expression analysis and WGCNA. Through differential expression analysis, we identified 264 DEGs distinguishing brucellosis patients from healthy controls. Furthermore, immune infiltration analysis (**Figure 2**) uncovered distinct perturbations in immune cell composition among brucellosis patients, characterized by increased proportions of CD8^+^ T cells, T follicular helper cells, and resting NK cells, alongside decreased proportions of naive B cells, naive CD4^+^ T cells, resting mast cells, and centroblasts. Notably, previous studies have demonstrated that brucellosis is associated with T lymphocyte immunodeficiency (20): compared to healthy controls, both acute and chronic brucellosis patients exhibit a significant reduction in CD4^+^ T cells and a marked increase in CD8^+^ T cells. Zheng et al. (21) further confirmed these findings, reporting significantly decreased CD4^+^ T cell proportions and elevated CD8^+^ T cell proportions in brucellosis patients relative to healthy subjects, and highlighting that the balance of CD4^+^/CD8^+^ T cells may serve as a critical determinant of brucellosis progression. Our immune infiltration results are consistent with these prior observations, reinforcing the reliability of our findings.

Previous studies have demonstrated that the proportion of Th1 cells in peripheral blood of acute brucellosis patients is significantly higher than that in chronic patients, while the levels of Th2 cells is markedly elevated in chronic patients compared to acute cases—indicating a dominant Th2-type immune response during chronic infection (22, 23). As illustrated in the box plot (**Figure 4**), the analysis of this study reveals that unique key genes in the blood can not only distinguish brucellosis patients from non-patients but also differentiate between acute and chronic infections. This indicates that Brucella participates in the host’s immune response and cellular damage during infection. The enrichment analysis results revealed that the genes under investigation are predominantly implicated in key immune-related processes, including the regulation of T cell activation and differentiation, leukocyte-cell adhesion, lymphocyte differentiation, and the negative regulation of immune responses. Functionally, these genes are involved in critical biological activities such as immune cell activation, cytokine receptor binding, MHC protein interaction, and cytokine binding. Additionally, they contribute to the formation of cellular components such as the outer side of the plasma membrane, pattern-recognition receptor complexes, secretory granule membranes, and the T cell receptor complex. Furthermore, these genes exhibit significant associations with pathways and processes central to immune function, including the differentiation of Th17, Th1, and Th2 cells, hematopoietic cell lineage development, inflammatory bowel disease pathogenesis, factor-receptor interactions, and T cell receptor signaling pathways. We hypothesize that potential biomarkers exist at the intersection of differentially expressed genes and immune - related key genes. Through a Venn diagram, we initially identified 14 key genes that may play a central role in the pathogenesis and progression of brucellosis.

The ever-expanding scale and inherent complexity of biological data have driven the increasing use of machine learning in biology to establish informative and predictive models for potential biological processes. Machine learning is particularly noteworthy for its ability to make accurate predictions in the absence of experimental data, thereby guiding future research endeavors (24). Therefore, in order to further identify key biomarkers from the 14 overlapping genes, various machine learning methods were applied. Ultimately, six genes were pinpointed: RTP5, KIF19, CDKN2A, RCAN2, GLB1L3, and IL12RB2. These genes may serve as crucial biomarkers for brucellosis. Using the original gene expression levels as predictive samples, the ROC curve demonstrated that these genes exhibited strong diagnostic performance, indicating their potential as biomarkers. KIF19 is a member of the kinesin superfamily (25). Kinesin family members play crucial roles in microtubule length regulation. Microtubules, a key component of the actin cytoskeleton, are involved in intracellular transport, facilitating the directed transport of various cargoes, including organelles, protein complexes, and mRNA. Among its related pathways are Golgi-to-ER retrograde transport and Vesicle-mediated transport. Upon entry into host cells, the Brucella reside in acidified phagosomal compartments known as endosomal Brucella containing vacuoles (eBCVs). eBCV continuously interacts with the endoplasmic reticulum (ER) structure and the conserved oligomeric Golgi (COG) complex, enabling the transport of COG-dependent retrograde Golgi vesicles to BCVs, thereby acquiring Golgi-derived membrane structures to form rBCV (26). Brucella’s intracellular circulation and survival in host cells rely on Golgi vesicular transport. Given that KIF19 is involved in the directional transport of various substances within cells, we hypothesize that Brucella invasion of the host may induce changes in KIF19, though the specific regulatory mechanism remains unclear. RCAN2 interacts with and inhibits calcineurin activity. The calcineurin signaling pathway plays a crucial role in multiple physiological processes, including cardiac hypertrophy (27), T cell activation (28), skeletal muscle cell differentiation and fiber type switching, as well as synaptic plasticity and neurotransmission (29). The second step of *Brucella* infection involves activating certain GTPases to enter macrophages via traditional zippering phagocytosis (26). The calcineurin signaling pathway is involved in this process. Budak F et al. (30) sorted CD4^+^ T cells from peripheral blood samples of patients with acute brucellosis, chronic brucellosis, and healthy controls, and screened more than 2,000 miRNAs. Compared with acute cases, 28 miRNAs exhibited significant changes in expression levels in chronic cases, and all these miRNAs had higher expression levels in chronic cases than those in the control group, indicating that these miRNAs play a potential role in the development of chronic brucellosis. They suggested that these miRNAs could serve as biomarkers for judging the transition of the disease to chronicity. The 28 miRNAs include hsa-miR-6511b-3p, hsa-miR-6511a-3p, hsa-miR-4436b-5p, and hsa-miR-129-1-3p, which have regulatory relationships with RCAN2 analyzed in our study (**Figure 6F**). This also suggests that changes in RCAN2 may be critical for the transition of brucellosis from the acute phase to the chronic phase. The main function of RTP5 (receptor transporter protein 5) is involved in biological processes such as protein membrane insertion and targeting to the cell membrane (31), and studies have also suggested that it participates in the formation of inflammation. The process of Brucella immune escape is as follows: after the bacteria enter host cells, they form eBCVs, which gradually interact with intracellular biomembranes to form rBCVs; following massive bacterial proliferation, autophagosomal BCVs (aBCVs) are generated and then released into the intercellular space (26). The occurrence of this series of processes requires vesicular transport and cell membrane fusion; therefore, we speculate that changes in RTP5 are critical for the early adhesion, survival, and transport of Brucella in the host. The GLB1L3 gene encodes beta-galactosidase beta 1-like 3 (32), which is involved in several important biological metabolic processes, such as carbohydrate metabolic processes and the regulation of beta-galactosidase activity (33). The regulation of its metabolic system enables Brucella to take advantage of the metabolic pathways and intermediates provided by the host to adapt to various environmental conditions in the host cell. Some strains of B. abortus, B. melitensis, and B. suis use galactose as the sole carbon source when tested on the vitamin and mineral based medium (34). Studies by Budak F et al. (30) have suggested that the differentially expressed miRNAs between acute and chronic brucellosis include hsa-miR-4486 and hsa-miR-766-3p, which have regulatory relationships with GLB1L3 analyzed in our study (**Figure 6F**). We hypothesize that alterations in GLB1L3 may reflect the capacity of Brucella’s metabolically specialized machinery for intracellular survival to more efficiently acquire and utilize nutrients across all phases of the infection cycle, thereby indirectly implying that Brucella has achieved successful adaptive colonization within the host. The ability to regulate its metabolism is also one of the keys to the successful adaptation of Brucella *in vivo*. Metabolic systems adapted to intracellular survival can make better use of nutrients at all stages of the infectious cycle (35). CDKN2A (Cyclin Dependent Kinase Inhibitor 2A) is a protein-coding gene involved in cell cycle regulation and apoptosis. Regulating the macrophages’ apoptosis is also one of the strategies used by Brucella to achieve intracellular persistence. By regulating the apoptosis of these host cells, especially macrophages, Brucella can reduce the bactericidal ability of immune cells (26). Liu and his colleagues (36) defined the role of miR-125b-5p-mediated A20 (TNFAIP3) regulation in macrophages activation of B. abortus-infection. According to their findings, reduced miR-125b-5p expression leads to increase in the A20 protein level, suppress the activation of NF-kB and leads to intracellular bacterial survival. Zinc finger protein A20 is a dual inhibitor of macrophage activation and apoptosis, both promoting apoptosis and acting as an anti-apoptotic protein (37). The miR-125b-5p has a regulatory relationship with CDKN2A analyzed in our study (**Figure 6F**). Changes in CDKN2A regulate changes in the cell cycle, which may indirectly reflect Brucella’s ability to evade the host’s immune attack and persist intracellularly. IL12RB2 is a receptor for interleukin-12 (IL-12) that promotes the proliferation of T cells and natural killer (NK) cells. By significantly enhancing the production of interferon-γ (IFN-γ), it induces the differentiation of T cells into the Th1 phenotype (38). During the initial stage of Brucella infection in mice, the host response developed resembles the T helper 1 (Th1) type, with production of IFN-γ by Th1 and natural killer cells, as well as production of IL-12 and tumor necrosis factor α (TNF-α) by infected macrophages (39). IFN-γ mediated Th1 immune response is essential for Brucella clearance. Changes in IL12RB2 may indicate early-stage brucellosis infection. The six genes identified above have been implicated in complex cellular and inflammatory immune responses within organisms, consistent with our enrichment analysis results. These findings may contribute to understanding the pathological processes of brucellosis and provide valuable insights into potential diagnostic biomarkers or therapeutic targets for brucellosis.

Our study has certain limitations that must be acknowledged. While we identified potential specific biomarkers capable of distinguishing between brucellosis and healthy patients, as well as between acute and chronic cases of brucellosis, these findings have not yet been validated in a larger patient cohort. Future research should prioritize the validation of these biomarkers across more extensive patient populations and conduct longitudinal studies to investigate the changes in biomarker expression throughout the disease progression and in response to treatment. Ultimately, the identification of reliable and specific biomarkers for brucellosis is a crucial step toward the development of effective diagnostic and treatment strategies for this neglected disease.

In summary, our study identified six genes—RTP5, KIF19, CDKN2A, RCAN2, GLB1L3, and IL12RB2—as potential biomarkers for brucellosis. The ROC curve analysis indicated that these genes exhibit strong diagnostic performance. Moreover, they are significantly associated with various immune processes, including the B cell receptor signaling pathway, cell cycle regulation, hematopoietic cell lineage, leukocyte endothelial migration, the proteasome, and Th1/Th2 cell differentiation. Consequently, these six mRNAs hold promise as novel biomarkers for brucellosis and present opportunities for the development of innovative immunotherapeutic strategies. 

## Data Availability

Publicly available datasets were analyzed in this study. This data can be found here: http://www.ncbi.nlm.nih.gov/geo/query/acc.cgi?acc=GSE69597.
